# Pulling a Ligase out of a “HAT”: pCAF Mediates Ubiquitination of the Class II Transactivator 

**DOI:** 10.1155/2017/8093813

**Published:** 2017-02-12

**Authors:** Julie E. Morgan, Susanna F. Greer

**Affiliations:** Division of Cellular Biology and Immunology, Department of Biology, Georgia State University, Atlanta, GA 30302, USA

## Abstract

The Class II Transactivator (CIITA) is essential to the regulation of Major Histocompatibility Class II (MHC II) genes transcription. As the “master regulator” of MHC II transcription, CIITA regulation is imperative and requires various posttranslational modifications (PTMs) in order to facilitate its role. Previously we identified various ubiquitination events on CIITA. Monoubiquitination is important for CIITA transactivity, while K63 linked ubiquitination is involved in crosstalk with ERK1/2 phosphorylation, where together they mediate cellular movement from the cytoplasm to nuclear region. Further, CIITA is also modified by degradative K48 polyubiquitination. However, the E3 ligase responsible for these modifications was unknown. We show CIITA ubiquitination and transactivity are enhanced with the histone acetyltransferase (HAT), p300/CBP associated factor (pCAF), and the E3 ligase region within pCAF is necessary for both. Additionally, pCAF mediated ubiquitination is independent of pCAF's HAT domain, and acetylation deficient CIITA is K48 polyubiquitinated and degraded in the presence of pCAF. Lastly, we identify the histone acetyltransferase, pCAF, as the E3 ligase responsible for CIITA's ubiquitination.

## 1. Introduction

Major Histocompatibility Class II (MHC II) genes are essential for the initiation of adaptive immune responses to extracellular pathogens; thus their expression and activation are of critical importance and are tightly regulated [1–3]. Coordinated orchestration of multiple proteins accomplishes transcription of MHC II; however, one protein in particular, known as the “master regulator” of MHC II genes, the Class II Transactivator, is particularly important [4–7]. In addition to CIITA, various other chromatin-remodeling enzymes are required for the “opening” of the MHC II promoter, thus allowing the transcriptional machinery to bind. In particular, two histone acetyltransferases (HATs), the CREB binding protein (CBP/p300) and p300/CBP associated factor (pCAF), are recruited to the MHC II promoter where they assist in the remodeling of chromatin which occurs before and in the presence of CIITA [8, 9].

CIITA is 1130 amino acid protein and is dynamically regulated through an intricate series of posttranslational modifications (PTMs) [10]. PTMs on CIITA include phosphorylation, ubiquitination, and acetylation [11–18]. These modifications precisely regulate CIITA's location, function, and stability within the cell and increase CIITA activity at the MHC II promoter [8, 10, 13–15, 19–22]. HATs including pCAF and CBP are responsible for acetylation of CIITA at lysine(s) (K) 141 and 144 [14]. It has further been shown that acetylation plays important roles in the ubiquitination of CIITA [13, 14]. Located at the N-terminal region of pCAF, lies a domain containing ubiquitin E3 ligase activity [23]. Ubiquitination requires three enzymes: an E1 activating enzyme, an E2 conjugating enzyme, and E3 ligase, which is responsible for the ligation of ubiquitin onto a substrate in conjunction with the E2 [24]. Previously pCAF's intrinsic ubiquitination domain was identified and shown to play a role in the ubiquitination and stability of the critical cell cycle protein, human double minute 2 (the human ortholog of Mdm2) [23, 25], and Gli1, a transcription factor that mediates hedgehog signaling [26]. Thus, pCAF is not only HAT, but also ubiquitin E3 ligase. Presently, pCAF is shown to ubiquitinate only a few substrates: Hdm2, Gli1, and itself [23, 25, 26]. As pCAF is known to affect the activity of many transcription factors and cofactors through its HAT activities, it is likely that pCAF also has additional targets for its ubiquitin E3 ligase activities. As CIITA has previously been shown to be a substrate for pCAF's HAT activity and observations have been made of CIITA's increased ubiquitination in the presence of pCAF [13], we sought to determine if pCAF was potential E3 ligase for CIITA.

We hypothesized pCAF is playing a novel role as ubiquitin E3 ligase for CIITA in addition to its traditional role as HAT. We show here that both CIITA transactivity levels and global ubiquitination (all ubiquitin types) significantly decline in the absence of the pCAF E3 ligase domain. Further, we demonstrate CIITA ubiquitination does not rely on the HAT domain of pCAF. Acetylation null CIITA mutants lacking the signal to become nuclear bound are ubiquitinated in a K48 linked fashion leading to degradation. In vitro ubiquitination assays confirm pCAF's ability to facilitate CIITA ubiquitination. Lastly, we identify that CIITA mono, K63, and, K48 linked ubiquitination are mediated by pCAF in vivo. These results demonstrate pCAF's capacity to facilitate various topologies of CIITA ubiquitination. These results indicate that pCAF, via its E3 ligase activity, plays additional important roles in the regulation of CIITA activity and thus in regulating the expression of MHC II genes. Further, identification of the E3 ligase responsible for ubiquitination of CIITA is critical for gaining added understanding of CIITA regulation by PTMs. Identifying enzymes responsible for these PTMs allows for valuable insight into the regulation of the adaptive immune response and for the identification of potential therapeutic targets.

## 2. Materials and Methods

### 2.1. Cell Culture

COS cells (Monkey fibroblast) from ATCC (Manassas, VA) were maintained using high-glucose Dulbecco's modified Eagle medium (DMEM) (Mediatech, Inc., Herndon, VA) supplemented with 10% fetal bovine serum, 50 units/mL of penicillin, 50 *μ*g/mL of streptomycin, and 2 mM of L-glutamine. Cells were maintained at 37° with 5% CO_2_.

### 2.2. Plasmids and Purified Proteins

Flag-K141R, K144R, K141/144R, and Myc-CIITA were kindly provided by Dr. Jenny Ting. Flag-pCAF was a generous gift from Drs. Linares et al. [23]. Myc-pCAF was subcloned into Myc tagged pCMV-3 using the EcoR1 restriction site. HA-K48 Ub and K63 Ub were a gift from Dr. Ted Dawson. Both, HA-K48 and K63 ubiquitin have all internal lysine residues of ubiquitin mutated to arginine except K48 or K63, allowing polyubiquitination to only occur on those lysine residues. The HLA-DRA luciferase reporter construct was described previously [27]. The E1 activating enzyme UBE1 (Boston Biochem, Boston, MA), E2 conjugating enzyme UbcH5b (Boston Biochem), Flag ubiquitin (Boston Biochem), Hdm2 (Boston Biochem), and His-pCAF (Proteinone, Rockville, MD) were all obtained commercially.

### 2.3. GST-Protein Production and Purification

BL21 star (DE3) competent cells (Invitrogen, Carlsbad, CA) were transformed with pGEX constructs. Transformed colonies were selected and inoculated in 5 mL LB supplemented with AMP and bacteria were allowed to grow overnight at 37°. One mL preps was added to 100 mL fresh LB supplemented AMP and bacterial were allowed to grow for three and a half hours at 37° to OD_600_, 0.8. IPTG was added to induce expression. Cells were centrifuged and the pellet was washed with chilled PBS and centrifuged again. The cell pellet was frozen for one hour at −80°; pellet was allowed to thaw on ice and was resuspended in buffer A (PBS + 1% Triton-X100 + 0.1 M NaCl + Protease Inhibitor (Roche)). Cells were sonicated on ice and were centrifuged to obtain the soluble fraction. The insoluble fraction was then resuspended in buffer B (buffer A + 25% (w/v) sucrose) and the mixture was centrifuged at 20,000 rpm for 20 minutes. The supernatant was then collected as the insoluble fraction. Solubilization and refolding of inclusion bodies were performed in 8 M urea + 5 mM DTT to dissolve the pellet. The protein-urea mixture was then dialyzed in PBS at 4° for two days. GST-CIITA protein was added to a Glutathione Resin column and the protein was eluted in 10 mM glutathione elution buffer (0.154 g reduced glutathione dissolved in 50 mL of 50 mM Tris-HCL, pH 8.0). GST-CIITA, flow through, wash, and elutes were analyzed by SDS-PAGE and then stained with Coomassie. Elutes were dialyzed to remove free glutathione.

### 2.4. Coimmunoprecipitations

COS cells were plated at cell density of 8 × 10^5^/10 cm on tissue culture plates. Cells were transfected using GeneJuice (Merck Millipore, Darmstadt, Germany) as indicated with 5 *μ*g of Myc-CIITA, Flag-pCAF, Flag-K141R, K144R, K141/144R CIITA, HA-K48 Ub, K63 Ub, HA-mono Ub, or pCDNA control. Twenty-four hours after transfections, cells were lysed in 1% NP40 buffer supplemented with EDTA-free protease inhibitors (Roche) on ice. Lysates were centrifuged, normalized for protein concentration, and precleared with Mouse IgG (Sigma-Aldrich) and Protein G (Thermo Fisher) followed by immunoprecipitation with either EZ view anti-c Myc affinity gel beads (Sigma-Aldrich) or with anti-Flag M2 affinity gel (Sigma-Aldrich). Immune complexes were denatured with Laemmli buffer, boiled, and separated by SDS-PAGE gel electrophoresis. Gels were transferred to nitrocellulose and were individually immunoblotted with anti-Myc (Abcam, Cambridge, MA), anti-Flag (Sigma-Aldrich), antiubiquitin (Life Sensors, Malvern, PA), anti-K48 ubiquitin (Cell Signaling, Danvers, MA), anti-K63 ubiquitin (Millipore), or with anti-GST (Abcam, Cambridge, MA). HRP conjugates were detected using HyGlo Chemiluminescent substrate (Denville). Protein normalization and equal loading were determined in lysates.

### 2.5. Luciferase Reporter Assays

COS cells were plated at 5 × 10^4^ cells/well density (70% confluency). Following adhesion, cells were cotransfected as indicated with HLA-DRA, Renilla, pcDNA, Myc-CIITA, and Flag-pCAF using GeneJuice (Merck Millipore, Darmstadt, Germany) according to the manufacturer's protocol. Twenty-four hours following transfections, cells were lysed with 1x Passive lysis buffer (Promega, Madison, WI) supplemented with EDTA-free protease inhibitor (Roche). Dual luciferase assays were performed using the Lmax II_384_ (Molecular Devices, Sunnyvale, CA) according to the manufacturer's instructions. Luciferase readings were normalized to Renilla readings for protein normalization.

### 2.6. In Vitro Ubiquitination Assay

The CIITA in vitro ubiquitination assay was carried out in 150 *μ*L of reaction mixture containing 40 mM Tris-HCL (pH 7.5), 5 mM MgCl_2_, 2 mM dithiothreitol, 1 mM Creatine Phosphate, 2 mM ATP, 400 ng of Recombinant GST-CIITA (substrate), 400 ng of GST-Hdm2 (Boston Biochem), 400 ng Recombinant His-pCAF (E3 ligase candidate) (Proteinone Rockville, MD), 500 ng Flag ubiquitin (Boston Biochem, Boston, MA), 200 ng E1 activating enzyme, UBE1 (Boston Biochem), 200 ng E2 conjugating enzyme, and UbcH5b (Boston Biochem). All components were added and incubated at 37°C for 60 minutes and were analyzed via SDS-PAGE. Ubiquitination was detected by immunoblot using Flag antibody (Sigma), and CIITA ubiquitination was verified with GST (Abcam), and pCAF was verified with monoclonal *α* pCAF antibody (Santa Cruz). Verification of Hdm2 ubiquitination was detected using GST (Abcam).

## 3. Results

### 3.1. CIITA and pCAF Coimmunoprecipitate

**Figure 1:**
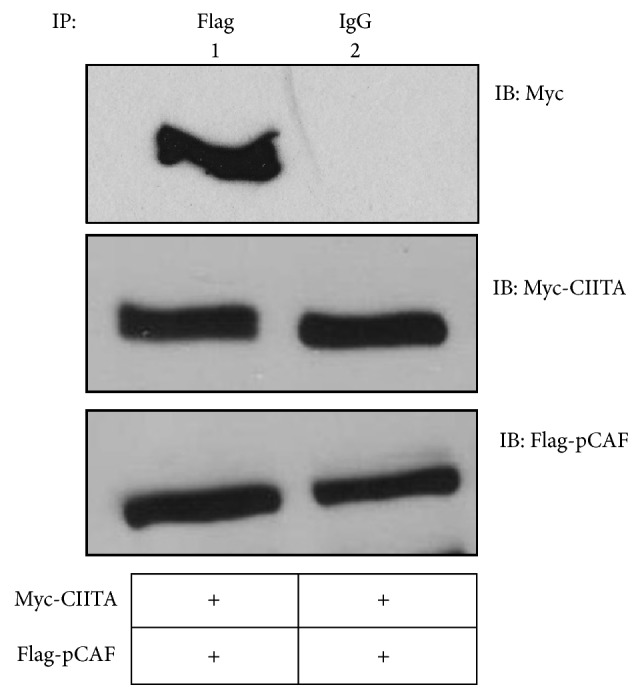
CIITA associates with the E3 ligase pCAF.* Coimmunoprecipitation of CIITA and pCAF*. COS cells were cotransfected with Myc-CIITA and Flag-pCAF. Cells were harvested, lysed, precleared, and immunoprecipitated with anti-Flag and anti-Mouse IgG. Western blots were performed and immunoprecipitated samples were immunoblotted using anti-Myc antibodies. Lysate controls demonstrate expressions of Myc-CIITA and Flag-pCAF. Data shown are cropped images from one immunoprecipitation gel and one lysate gel and are representative of three individual experiments.

CIITA and pCAF previously have been shown to associate [14], and pCAF is known to acetylate CIITA on lysines (K) 141 and 144. These residues lie within a nuclear localization signal (NLS) region and acetylation is necessary to shuttle CIITA to the nucleus. Once pCAF acetylates CIITA, CIITA accumulates in the nucleus, where it binds to the enhanceosome complex at the MHC II promoter [14] and drives MHC II transcription. To confirm previous findings, we conducted coimmunoprecipitation assays to verify interactions between WT CIITA and WT pCAF. Lane one indicates association of WT Myc-CIITA and WT Flag-pCAF through coimmunoprecipitation analysis (Figure 1, top panel, lane one).

### 3.2. pCAF's E3 Ligase Domain Is Necessary for CIITA Transactivity

**Figure 2:**
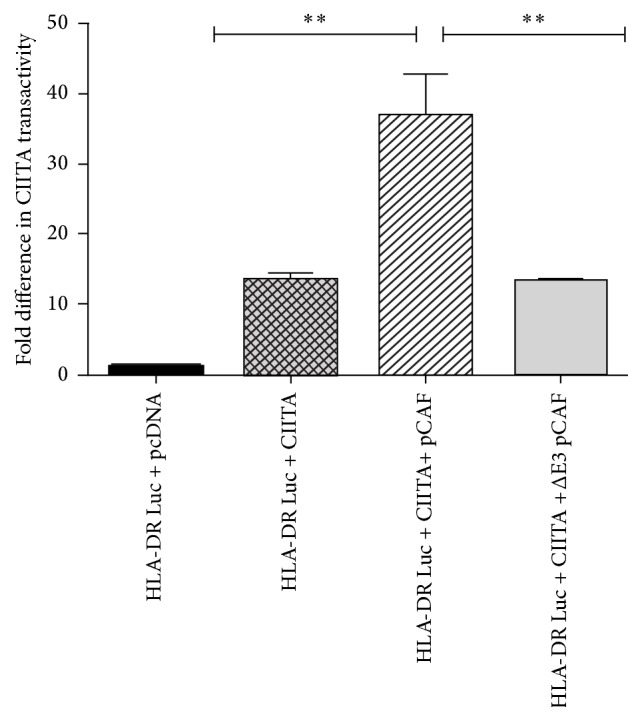
The pCAF E3 ligase domain is necessary for enhanced CIITA transactivity. CIITA transactivation increases in the presence of expressed WT pCAF.* Reporter Assay*. COS cells were transfected as indicated with the MHC II HLA-DR Luc reporter construct, Renilla, CIITA, pCAF, and Δ E3 pCAF. Luciferase assays were performed in triplicate in three independent experiments; data are presented as fold increase in luciferase activity. Results are standardized to Renilla values and represent the mean ± SD, *∗∗* < 0.01.

While pCAF is known primarily for its HAT role, pCAF is also considered to be an ubiquitination factor with intrinsic E3 ligase capabilities [23, 26]. Interestingly, pCAF does not have any homology to other known E3 ligases. Linares et al., performed a series of deletion mutations and identified a region (amino acids 121–242) that possesses E3 ligase capability [23]. Previous reports suggest pCAF is able to mediate both acetylation and ubiquitination of the same target [13, 23, 26, 28]. Thus, we next wanted to determine if pCAF's E3 ligase domain is necessary for CIITA's increased transactivity. Levels of transactivity were determined using a dual luciferase reporter assay. CIITA cotransfected with WT pCAF leads to a 2-fold increase in CIITA transactivity and ability to drive MHC II transcription; however, deletion of the E3 ligase domain of pCAF leads to a significant decrease in CIITA transactivity levels (Figure 2).

### 3.3. E3 Ligase Domain Deficient pCAF Is Unable to Ubiquitinate CIITA

**Figure 3:**
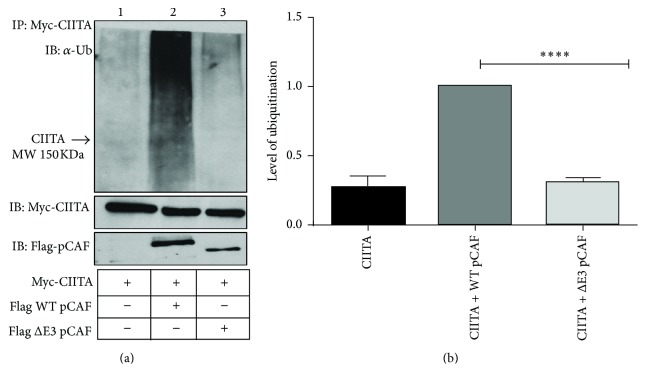
CIITA ubiquitination depends on the E3 ligase domain of pCAF. (a) The E3 ligase null mutant pCAF negates CIITA ubiquitination. In vivo* ubiquitination assay.* COS cells were cotransfected with Myc-CIITA, Flag-pCAF, and Flag-pCAF Δ E3 ligase mutants as indicated. Eighteen hours following transfections, cells were harvested, lysed, precleared, and immunoprecipitated with the anti-Myc antibody. Western blots were performed and immunoprecipitated samples were immunoblotted using antiubiquitin antibodies. Lysate controls (bottom two panels) demonstrate expression of Myc-CIITA, Flag-pCAF, and Flag-pCAF Δ E3. Data shown are cropped images from one immunoprecipitation gel and one lysate gel and are representative of three independent experiments. (b) Densitometry and quantification of data in Figure 3(a). Densitometry was performed on three independent experiments, mean ± SD, *∗∗∗∗* < 0.0001. Area for densitometry analysis was selected from CIITA's molecular weight of 150 KDa as labeled and above.

We next investigated if CIITA ubiquitination would be impaired or altered in the absence of the E3 ligase domain of pCAF. pCAF contains an autoubiquitination domain that is able to mediate self-ubiquitination but has not been shown to be involved in ubiquitination of other substrates [23]. To determine if the E3 ligase domain is necessary for facilitating CIITA ubiquitination, we performed an in vivo ubiquitination assay. WT-Myc-CIITA, WT-Flag-pCAF, or the ΔE3 pCAF mutant were cotransfected into COS cells. Ubiquitination levels of WT CIITA cotransfected with WT pCAF show a significant increase over those of WT CIITA transfected alone (Figure 3, compare lanes 1 and 2). However, the ubiquitination levels of CIITA cotransfected with the ΔE3 pCAF mutant show levels of ubiquitination that are significantly decreased when compared to those of CIITA cotransfected with WT pCAF (Figure 3, compare lanes 2 and 3). These data support that the E3 ligase domain of pCAF is important for CIITA ubiquitination and is involved in mediating CIITA ubiquitination.

### 3.4. pCAF Facilitates CIITA Ubiquitination Independent of Its HAT Domain

**Figure 4:**
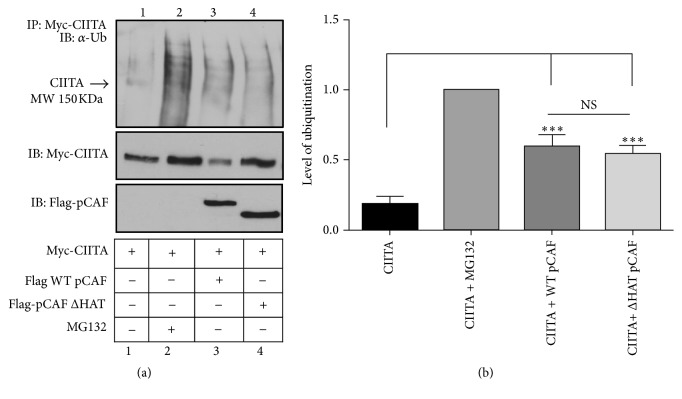
pCAF facilitates CIITA ubiquitination independent of its HAT domain. (a) CIITA is ubiquitinated by pCAF. In vivo* ubiquitination assay.* COS cells were cotransfected as indicated with Myc-CIITA, Flag-pCAF, and Flag-pCAF Δ HAT mutant. Eighteen hours following transfections, cells were harvested, lysed, precleared, and immunoprecipitated with the anti-Myc antibody. Western blots were performed and immunoprecipitated samples were immunoblotted using antiubiquitin antibodies. Lysate controls (bottom two panels) demonstrate expression of Myc-CIITA, Flag-pCAF, and Flag-pCAF Δ HAT. Data shown are cropped images from one immunoprecipitation gel and one lysate gel and are representative of three independent experiments. (b) Densitometry and quantification of data in (a). Densitometry was performed on three independent experiments, mean ± SD, *∗∗∗* < 0.001. Area for densitometry analysis was selected from CIITA's molecular weight of 150 KDa as labeled and above.

pCAF is a well-known HAT and is involved in many aspects of CIITA and MHC II regulation [14, 29, 30]. pCAF assists in remodeling the chromatin structure of the MHC II promoter where it acetylates histones H3 and H4 [30] and also regulates CIITA's nuclear relocation by acetylating CIITA K141 and K144, leading to increased activation of CIITA and increased expression of MHC II [14]. To determine if pCAF's role in ubiquitination of CIITA was independent of pCAF's HAT activities, we performed in vivo ubiquitination assays using WT CIITA and HAT deletion mutant of pCAF. In this assay, expression of WT CIITA alone indicates low level ubiquitination (Figure 4(a), lane 1), and ubiquitination levels significantly increase when WT pCAF is overexpressed (Figure 4(a), lane 3). However, in the absence of the pCAF HAT domain, there is no measureable difference in CIITA ubiquitination levels when CIITA ubiquitination is compared to that generated in the presence of WT pCAF (Figure 4(a), compare lanes 3 and 4). Thus, we conclude that pCAF's ability to ubiquitinate CIITA is independent of the pCAF HAT domain.

### 3.5. pCAF Associates with CIITA Acetylation Null Mutants

**Figure 5:**
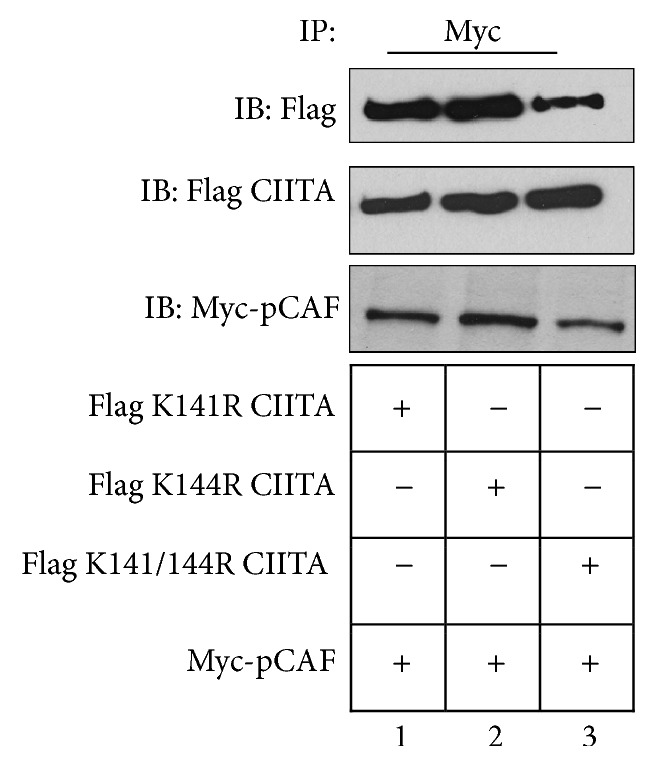
Acetylation null CIITA mutants coimmunoprecipitate with pCAF.* Coimmunoprecipitation of acetylation null CIITA mutants with pCAF.* COS cells were cotransfected with Myc-CIITA, K141R CIITA, K144R CIITA or K141/144R CIITA, and Flag-pCAF. Cells were harvested, lysed, precleared, and immunoprecipitated with anti-Myc or Mouse IgG antibodies as indicated. Western blots were performed and immunoprecipitated samples were immunoblotted using anti-Myc antibodies. Lysate controls demonstrate expression of Myc-CIITA and acetylation null mutants and Flag-pCAF (bottom two panels). Data shown are cropped images from one immunoprecipitation gel and one lysate gel and are representative of 3 individual experiments.

As pCAF acts in a dual way as both HAT and ubiquitin ligase, and as acetylation drives CIITA nuclear import, we next determined if acetylation of CIITA is necessary for CIITA ubiquitination mediated by pCAF. To begin to investigate the relationship between acetylation and ubiquitination on CIITA, we first wanted to identify if acetylation null CIITA mutants and pCAF are able to associate. Coimmunoprecipitation analysis indicates CIITA acetylation mutants deficient at K141R, K144R individually, or K141/144R double mutant sites all remain capable of interaction with pCAF (Figure 5).

### 3.6. pCAF Mediates K48 Linked Ubiquitination of Acetylation Null CIITA Mutants

**Figure 6:**
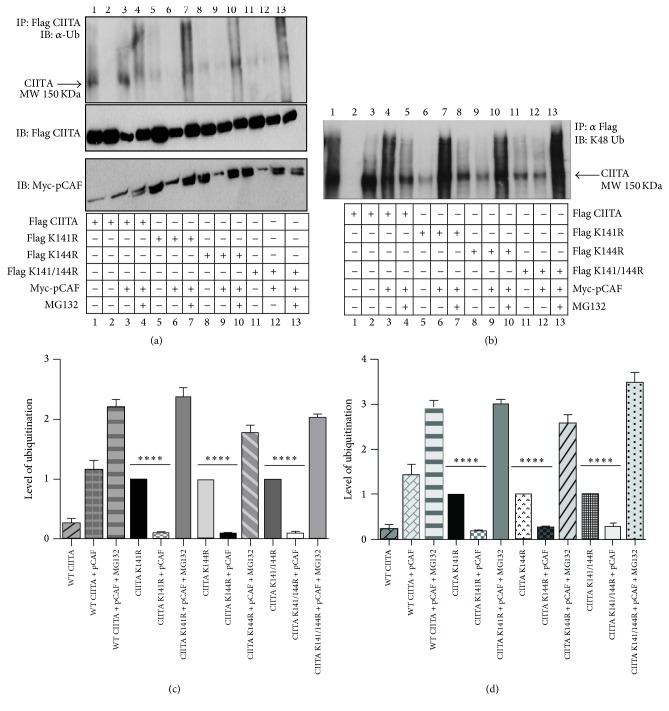
pCAF enhances ubiquitination of CIITA acetylation null mutants. (a) Acetylation null CIITA mutants have increased levels of degradative ubiquitination in the presence of pCAF. In vivo* ubiquitination assay*. COS cells were cotransfected with Flag-K141R, K144R, K141/144R CIITA, and Myc-pCAF. Eighteen hours following transfections, MG132 was added to indicated samples in order to inhibit the 26S proteasome. Cells were harvested, lysed, precleared, and immunoprecipitated with anti-Flag antibody (lane 2 negative control, immunoprecipitated with IgG). Western blots were performed and immunoprecipitated samples were immunoblotted using antiubiquitin antibodies. Lysate controls (bottom two panels) demonstrate expression of WT Flag CIITA, Flag-K141R, K144R, K141/144R, and Myc-pCAF. Data shown are cropped images from one immunoprecipitation gel and one lysate gel and are representative of three individual experiments. (b) CIITA acetylation null mutants have increased levels of K48 specific linkage ubiquitination in the presence of pCAF. 15 *μ*L from the same sample was immunoprecipitated with anti-Flag antibody and immunoblotted for anti-K48 specific ubiquitin antibody. (c) Densitometry and quantification of data in (a). Densitometry was performed on three independent experiments, mean ± SD, *∗∗∗∗* < 0.0001. (d) Densitometry and quantification of data in (b). Densitometry was performed on three independent experiments, mean ± SD, *∗∗∗∗* < 0.0001. Area for densitometry analysis was selected from CIITA's molecular weight of 150 KDa as labeled and above.

Acetylation at K141 and K144 of CIITA is critical in signaling the movement of CIITA from the cytoplasm to the nucleus [14]. To investigate pCAF's role in mediating acetylation independent ubiquitination, we utilized acetylation null mutants of CIITA. These CIITA mutants are incapable of being acetylated at K141 and K144 and thus likely targets for K48 linked polyubiquitination and degradation. Our results indicate K141R and K144R and K141/144R CIITA mutants display low levels of ubiquitination and the addition of pCAF yields significantly lower levels of detectable ubiquitination (Figure 6(a), top blot) as compared to WT CIITA coexpressed with pCAF (compare lanes 3, 6, 9, and 12). Further, lysate blots indicate CIITA transfections and show a decrease in CIITA protein expression (Figure 6(a), middle blot). These data suggest the lysine deficient CIITA is being degraded at greater rate with the overexpression of pCAF (Figure 6(a), compare lanes 5 and 6). Proteasome inhibition by MG132 indicates an accumulation of ubiquitinated CIITA when pCAF is present (Figure 6(a) lane 7), indicating the lack of ubiquitination smear seen in lane 6 is likely due to CIITA degradation. Further, the K144R mutant and the double K141/K144R mutants indicate similar trends, respectively (Figure 6(a) lanes 8–10 and 11–13). To further determine if pCAF mediates CIITA K48 linked ubiquitination, we took 15 uL of the same immunoprecipitation sample and immunoblotted it for K48 ubiquitination using specific antibodies recognizing K48 ubiquitination (Figure 6(b)). Similar trends were observed as seen in Figure 6(a), where CIITA acetylation mutants were ubiquitinated; however, when pCAF is introduced we again observe that ubiquitination diminishes and samples treated with proteasome inhibitor show accumulation of ubiquitinated CIITA. Densitometry assays indicate significant differences in ubiquitination levels in both global ubiquitination and K48 specific ubiquitination (Figures 6(c) and 6(d)).

### 3.7. In Vitro Ubiquitination Assays Indicate pCAF's Ability to Mediate Ubiquitination of CIITA

**Figure 7:**
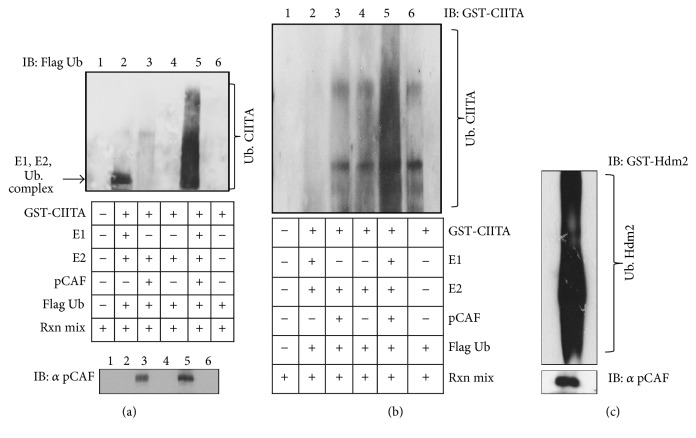
pCAF ubiquitinates CIITA in vitro. (a) Ubiquitination of CIITA by pCAF in vitro. Reaction components are as indicated and were performed as described in Material and Methods. Ubiquitination was detected with anti-Flag antibody (top panel); pCAF was detected using anti-pCAF antibody (bottom panel). (b) Ubiquitinated CIITA was further detected with anti-GST antibody. (c) Positive control demonstrating pCAF's ability to facilitate ubiquitination in vitro on Hdm2.

To elucidate if CIITA was a substrate for pCAF as E3 ligase, we conducted an in vitro ubiquitination assay using purified human recombinant proteins. As a positive control, we used GST-Hdm2, which has previously been seen to be ubiquitinated by pCAF [23] (Figure 7(c)). Purified human recombinant proteins E1 (UbA-1), E2 (UbCH5B), Flag ubiquitin, GST-WT CIITA, and His-WT pCAF (E3) were all used in the presence of a reaction mixture (see Materials and Methods). As shown in Figures 7(a) and 7(b), when all ubiquitination components (E1, E2, pCAF (E3), and ubiquitin) were present along with the substrate in question, CIITA ubiquitination occurs (Figures 7(a) and 7(b), lane 5); however in the absence of any of these components, ubiquitination did not occur. We immunoblotted both anti-Flag ubiquitin and anti GST-CIITA. These data reveal that CIITA is a substrate for the ubiquitin E3 ligase pCAF.

### 3.8. CIITA Mono, K63, and K48 Linked Ubiquitination Is Increased In Vivo with pCAF Expression

**Figure 8:**
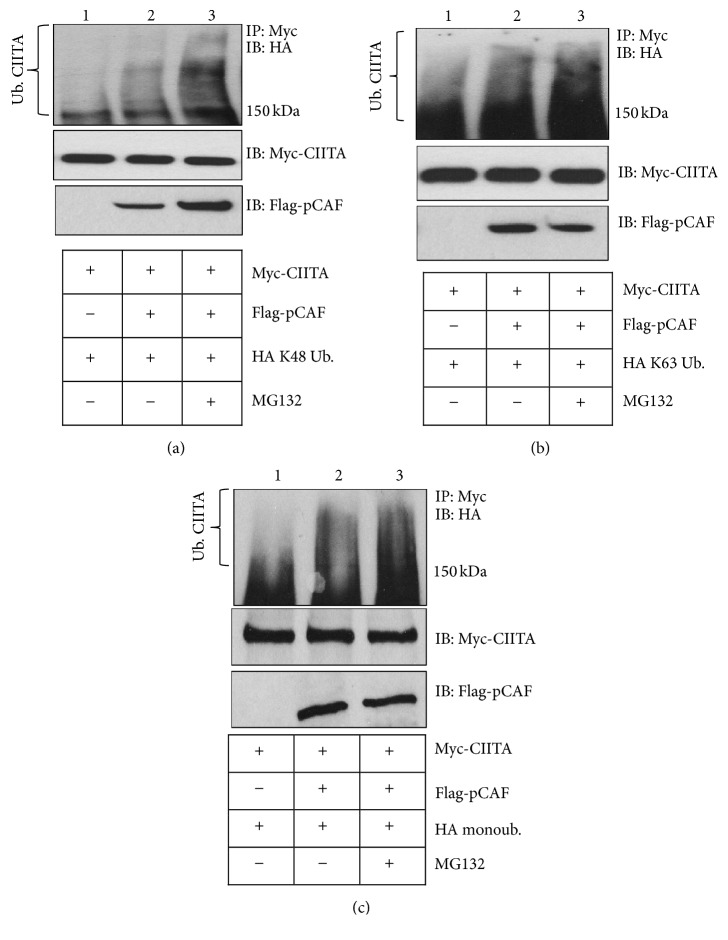
pCAF enhances K48, K63, and monoubiquitination of CIITA. (a)* In vivo ubiquitination assay*. COS cells were cotransfected with Myc-CIITA, Flag-pCAF, HA-K48 ubiquitin, HA-K63 ubiquitin, or HA-mono Ub. Eighteen hours following transfections, MG132 was added to indicated samples to inhibit the 26S proteasome in treated cells. Cells were harvested, lysed, precleared, and immunoprecipitated with anti-Myc antibody. Western blots were performed and immunoprecipitated samples were immunoblotted using anti-HA antibody. Lysate controls (bottom two panels) demonstrate expression of Myc-CIITA and Flag-pCAF. Data shown are cropped images from one immunoprecipitation gel and one lysate gel and are representative of three individual experiments.

We previously determined CIITA's ubiquitination status to include mono, K63, and K48 ubiquitination [10, 11]. Bhat et al. showed the three sites of monoubiquitination on CIITA are necessary for CIITA stability and for increased CIITA transactivity. Additionally, we previously demonstrated CIITA modified by K63 linked ubiquitination plays a role in CIITA movement from the cytoplasm to the nucleus. Knowing that several types of ubiquitination linkages modify CIITA, we wanted to determine if pCAF was able to facilitate all three types of CIITA ubiquitination. Previously, we demonstrated pCAF is able to mediate K48 linked CIITA ubiquitination in the absence of CIITA acetylation. To investigate if pCAF is able to mediate various ubiquitin linkages on CIITA, we conducted in vivo ubiquitination assays. We demonstrate CIITA monoubiquitination, K63, and K48 linked ubiquitination all increase in the presence of WT pCAF as compared to assays performed when WT CIITA is expressed alone (Figures 8(a), 8(b), and 8(c), compare lanes 1 and 2). When the proteasome inhibitor MG132 inhibits the 26S proteasome, all three forms of ubiquitinated CIITA accumulated indicating maximum ubiquitination levels (Figures 8(a), 8(b), and 8(c), lane 3). These data indicate pCAF's ability to mediate multiple forms of ubiquitination of CIITA.

## 4. Discussion

We sought here to identify the ubiquitin E3 ligase mediating CIITA ubiquitination. CIITA is known as the “master regulator” of MHC II genes. MHC II is critically important for proper presentation of extracellular pathogen to CD4^+^ T cells in adaptive immune responses. While MHC II is regulated at the level of transcription, CIITA is tightly regulated at the level of posttranslational modification. CIITA is heavily modified through a complex series of PTMs that dynamically regulate its location, function, stability, and activity.

Previous reports identify CIITA as being modified by mono, K63, and K48 linked ubiquitination [10, 11]. Ubiquitination of CIITA has been demonstrated as an essential posttranslational modification. Monoubiquitinated CIITA is more stable and active at the MHC II promoter [11, 13, 31]. K63 linked ubiquitination is also important as this particular ubiquitin linkage demonstrates enhanced crosstalk with phosphorylation and together these modifications are important for movement of CIITA from the cytoplasm to the nucleus [10]. Additionally, CIITA is modified by K48 linked ubiquitination, leading to recognition and degradation by the proteasome [11].

pCAF is a well-known histone acetyltransferase or HAT [32] and has previously been demonstrated to be recruited to and to activate transcription of the MHC II promoter via pCAF's HAT activities [2, 22]. pCAF must be localized to the MHC II promoter where pCAF cooperates with CIITA to drive MHC II transcription. The interaction of pCAF and CIITA is independent of pCAF's HAT domain [2, 8, 32]. We confirm here the addition of pCAF drives increased CIITA transactivation levels at the MHC II promoter (Figure 2).

Reports by several groups of pCAF's ability to act as E3 ubiquitin ligase, facilitating ubiquitination on targets such as Hdm2/Mdm2 and Gli1 [23, 26] where pCAF has also been shown to act as HAT, raised the possibility that pCAF could have dual enzyme activity on CIITA. We next sought to determine if pCAF was participating as E3 ligase and able to mediate CIITA ubiquitination. Levels of CIITA ubiquitination significantly increase in the presence of WT pCAF, while in the absence of the pCAF E3 ligase region, ubiquitination is abolished (Figure 3). The region of pCAF containing the E3 ligase domain does not conform to any known E3 ligase structures; many questions remain as to how pCAF may function as E3 ligase. Possible mechanism is many E3 ligases are regulated through autoubiquitination [33]. pCAF's autoubiquitination domain has previously been shown to not be involved in substrate ubiquitination but does promote self-ubiquitination [23]. E3 ligases also can be regulated by phosphorylation, leading to either activation or deactivation [34, 35]. pCAF is phosphorylated at tyrosine (Y) 729 and threonine (T) 731 and the role for phosphorylation has yet to be determined [36]; however, phosphorylation at these residues could be involved in pCAF's E3 ligase function.

We further show CIITA ubiquitination by pCAF is independent of pCAF's HAT domain (Figure 4(a)). When CIITA acetylation is blocked, CIITA is ubiquitinated by K48 linked polyubiquitination and subsequently degraded. This data is in line with previous reports indicating treatment with Trichostatin A (TSA) and HDAC1 which show reduced levels of CIITA ubiquitination [13]. By blocking the proteasome, we were able to visualize accumulated levels of ubiquitination (Figures 6(a) and 6(b)). These data indicate pCAF's ability to ubiquitinate independently of its HAT function.

In vitro analysis confirmed pCAF's role in ubiquitination of CIITA when all necessary ubiquitin components were available and pCAF as the E3; ubiquitination of CIITA occurred (Figures 7(a) and 7(b)). Further, In vivo ubiquitination assays revealed pCAF's ability to mediate multiple types of CIITA ubiquitination, including mono, K63, and K48 linked polyubiquitination (Figures 8(a), 8(b), and 8(c)). pCAF utilizes the E2 conjugating enzyme UbcH5b, which is capable of synthesizing ubiquitin chains containing all of the possible seven linkages (K6, 11, 27, 29, 33, 48, and 63) of ubiquitin [37].

Our study increases understanding of the regulation of the Class II Transactivator, thus leading directly to the molecular events, which contribute to the regulation of MHC II genes. Ubiquitination has been shown to be one of the many important and necessary PTMs, which regulate CIITA. Our previous identification of both mono and K63 linked ubiquitination provided valuable insight that ubiquitination regulates the stability, location, and activity of CIITA [10, 11]. Here we identify a novel substrate for the E3 ligase function of pCAF and identify pCAF's ability to mediate various ubiquitination moieties on CIITA. Future work will focus on understanding the regulation of pCAF as E3 ligase. pCAF does not contain homologous domains of other known E3 ligases and it remains unknown how enzymes with “dual” HAT and E3 ligase activity are regulated. Understanding the mechanism controlling each of these functions and how they are simultaneously regulated will be important to further understand the regulation of CIITA and the adaptive immune response [38].

## Abbreviations

CIITA: Class II Transactivator
MHC II: Major Histocompatibility Class II
PTMs: Posttranslational modifications
pCAF: p300/CBP associated factor
HAT: Histone acetyltransferase
Ub: Ubiquitin.

## References

[1] Abbas A. K., Janeway C. A. (2000). Immunology: improving on nature in the twenty-first century. *Cell*.

[2] Drozina G., Kohoutek J., Jabrane-Ferrat N., Peterlin B. M. (2005). Expression of MHC II genes. *Current Topics in Microbiology and Immunology*.

[3] Schnappauf F., Hake S. B., Camacho Carvajal M. M., Bontron S., Lisowska-Grospierre B., Steimle V. (2003). N-terminal destruction signals lead to rapid degradation of the major histocompatibility complex class II transactivator CIITA. *European Journal of Immunology*.

[4] Benoist C., Mathis D. (1990). Regulation of major histocompatibility complex class-II genes: X, Y and other letters of the alphabet. *Annual Review of Immunology*.

[5] Boss J. M., Jensen P. E. (2003). Transcriptional regulation of the MHC class II antigen presentation pathway. *Current Opinion in Immunology*.

[6] Chang C.-H., Fontes J. D., Peterlin M., Flavell R. A. (1994). Class II transactivator (CIITA) is sufficient for the inducible expression of major histocompatibility complex class II genes. *Journal of Experimental Medicine*.

[7] Fontes J. D., Kanazawa S., Nekrep N., Peterlin B. M. (1999). The class II transactivator CIITA is a transcriptional integrator. *Microbes and Infection*.

[8] Harton J. A., Zika E., Ting J. P.-Y. (2001). The histone acetyltransferase domains of CREB-binding protein (CBP) and p300/CBP-associated factor are not necessary for cooperativity with the class II transactivator. *The Journal of Biological Chemistry*.

[9] Zika E., Fauquier L., Vandel L., Ting J. P.-Y. (2005). Interplay among coactivator-associated arginine methyltransferase 1, CBP, and CIITA in IFN-*γ*-inducible MHC-II gene expression. *Proceedings of the National Academy of Sciences of the United States of America*.

[10] Morgan J. E., Shanderson R. L., Boyd N. H., Cacan E., Greer S. F. (2015). The class II transactivator (CIITA) is regulated by post-translational modification cross-talk between ERK1/2 phosphorylation, mono-ubiquitination and Lys^63^ ubiquitination. *Bioscience Reports*.

[11] Bhat K. P., Truax A. D., Greer S. F. (2010). Phosphorylation and ubiquitination of degron proximal residues are essential for class II transactivator (CIITA) transactivation and major histocompatibility class II expression. *The Journal of Biological Chemistry*.

[12] Greer S. F., Harton J. A., Linhoff M. W., Janczak C. A., Ting J. P.-Y., Cressman D. E. (2004). Serine residues 286, 288, and 293 within the CIITA: a mechanism for down-regulating CIITA activity through phosphorylation. *Journal of Immunology*.

[13] Greer S. F., Zika E., Conti B., Zhu X.-S., Ting J. P.-Y. (2003). Enhancement of CIITA transcriptional function by ubiquitin. *Nature Immunology*.

[14] Spilianakis C., Papamatheakis J., Kretsovali A. (2000). Acetylation by PCAF enhances CIITA nuclear accumulation and transactivation of major histocompatibility complex class II genes. *Molecular and Cellular Biology*.

[15] Tosi G., Jabrane-Ferrat N., Peterlin B. M. (2002). Phosphorylation of CIITA directs its oligomerization, accumulation and increased activity on MHCII promoters. *EMBO Journal*.

[16] Voong L. N., Slater A. R., Kratovac S., Cressman D. E. (2008). Mitogen-activated protein kinase ERK1/2 regulates the class II transactivator. *Journal of Biological Chemistry*.

[17] Wu X., Kong X., Chen D. (2011). SIRT1 links CIITA deacetylation to MHC II activation. *Nucleic Acids Research*.

[18] Wu X., Kong X., Luchsinger L., Smith B. D., Xu Y. (2009). Regulating the activity of class II transactivator by posttranslational modifications: exploring the possibilities. *Molecular and Cellular Biology*.

[19] Jabrane-Ferrat N., Nekrep N., Tosi G., Esserman L., Peterlin B. M. (2003). MHC class II enhanceosome: how is the class II transactivator recruited to DNA-bound activators?. *International Immunology*.

[20] Masternak K., Muhlethaler-Mottet A., Villard J., Zufferey M., Steimle V., Reith W. (2000). CIITA is a transcriptional coactivator that is recruited to MHC class II promoters by multiple synergistic interactions with an enhanceosome complex. *Genes & Development*.

[21] Sisk T. J., Nickerson K., Kwok R. P. S., Chang C.-H. (2003). Phosphorylation of class II transactivator regulates its interaction ability and transactivation function. *International Immunology*.

[22] Wright K. L., Ting J. P.-Y. (2006). Epigenetic regulation of MHC-II and CIITA genes. *Trends in Immunology*.

[23] Linares L. K., Kiernan R., Triboulet R. (2007). Intrinsic ubiquitination activity of PCAF controls the stability of the oncoprotein Hdm2. *Nature Cell Biology*.

[24] Ciechanover A. (1994). The ubiquitin-mediated proteolytic pathway: mechanisms of action and cellular physiology. *Biological Chemistry Hoppe-Seyler*.

[25] Wang X., Taplick J., Geva N., Oren M. (2004). Inhibition of p53 degradation by Mdm2 acetylation. *FEBS Letters*.

[26] Mazzà D., Infante P., Colicchia V. (2013). PCAF ubiquitin ligase activity inhibits Hedgehog/Gli1 signaling in p53-dependent response to genotoxic stress. *Cell Death and Differentiation*.

[27] Bhat K. P., Turner J. D., Myers S. E., Cape A. D., Ting J. P.-Y., Greer S. F. (2008). The 19S proteasome ATPase Sug1 plays a critical role in regulating MHC class II transcription. *Molecular Immunology*.

[28] Kass E. M., Poyurovsky M. V., Zhu Y., Prives C. (2009). Mdm2 and PCAF increase Chk2 ubiquitination and degradation independently of their intrinsic E3 ligase activities. *Cell Cycle*.

[29] Chou S.-D., Khan A. N. H., Magner W. J., Tomasi T. B. (2005). Histone acetylation regulates the cell type specific CIITA promoters, MHC class II expression and antigen presentation in tumor cells. *International Immunology*.

[30] Li G., Harton J. A., Zhu X., Ting J. P.-Y. (2001). Downregulation of CIITA function by protein kinase A (PKA)-mediated phosphorylation: mechanism of prostaglandin E, cyclic AMP, and PKA inhibition of class II major histocompatibility complex expression in monocytic lines. *Molecular and Cellular Biology*.

[31] Drozina G., Kohoutek J., Nishiya T., Peterlin B. M. (2006). Sequential modifications in class II transactivator isoform 1 induced by lipopolysaccharide stimulate major histocompatibility complex class II transcription in macrophages. *Journal of Biological Chemistry*.

[32] Zika E., Ting J. P.-Y. (2005). Epigenetic control of MHC-II: interplay between CIITA and histone-modifying enzymes. *Current Opinion in Immunology*.

[33] De Bie P., Ciechanover A. (2011). Ubiquitination of E3 ligases: self-regulation of the ubiquitin system via proteolytic and non-proteolytic mechanisms. *Cell Death and Differentiation*.

[34] Hunter T. (2007). The age of crosstalk: phosphorylation, ubiquitination, and beyond. *Molecular Cell*.

[35] Woelk T., Sigismund S., Penengo L., Polo S. (2007). The ubiquitination code: a signalling problem. *Cell Division*.

[36] Hornbeck P. V., Zhang B., Murray B., Kornhauser J. M., Latham V., Skrzypek E. (2015). PhosphoSitePlus, 2014: mutations, PTMs and recalibrations. *Nucleic Acids Research*.

[37] Li W., Ye Y. (2008). Polyubiquitin chains: functions, structures, and mechanisms. *Cellular and Molecular Life Sciences*.

[38] Morgan J. E. (2015). *Dynamic regulation of the class II transactivator by posttranslational modifications [dissertation]*.

